# The relationships between IBS and perceptions of physical and mental health—a Norwegian twin study

**DOI:** 10.1186/s12876-022-02340-8

**Published:** 2022-05-28

**Authors:** Julia Kutschke, Jennifer R. Harris, May-Bente Bengtson

**Affiliations:** 1 Vestfold Hospital Trust, Tønsberg, Norway; 2 Centre for Fertility and Health, The Norwegian Institute of Public Health, Oslo, Norway

**Keywords:** Twins, IBS, Self-reported physical health, Self-reported mental health, Hinderance of daily activity

## Abstract

**Background and aims:**

Poor quality of life is a main complaint among individuals with irritable bowel syndrome (IBS). Self-rated health (SRH) is a powerful predictor of clinical outcomes, and also reflects psychological and social aspects of life and an overall sense of well-being. This population-based twin study evaluates how IBS affects ratings of physical and mental health, and influences perceptions of hindrance of daily activity by physical or mental health. Further, we examine how IBS is related to these SRH measures.

**Methods:**

The sample included 5288 Norwegian twins aged 40–80, of whom 575 (10.9%) suffer from IBS. Hierarchical regressions were used to estimate the impact of IBS on perceptions of health, before and after accounting for other chronic physical and mental health conditions. Two dimensions of SRH, physical and mental, and two aspects of functional limitations, the extent to which physical or mental health interferes with daily activities, were included as outcomes in separate models. Co-twin control analyses were used to explore whether the relationships between IBS and the four measures of SRH are causal, or due to shared genetic or shared environment effects.

**Results:**

IBS was an independent predictor of poor self-rated physical health (OR = 1.82 [1.41; 2.33]), the size of this effect was comparable to that predicted by chronic somatic conditions. However, in contrast to somatic diseases, IBS was associated with the perception that poorer ratings of mental health (OR = 1.45 [1.02; 2.06]), but not physical health (OR = 1.23 [0.96; 1.58]), interfered with daily activity. The co‐twin control analyses suggest that causal mechanisms best explain the relationships between IBS with self-rated physical health and with hindrance of daily activities. In contrast, the relationship between IBS and self-rated mental health was consistent with shared genetic effects.

**Conclusion:**

IBS is predictive of poor self-rated physical health. The relationship between IBS and self-rated mental health is best explained by shared genetic effects which might partially explain why mental health interferes with daily activity to a larger degree among those with IBS.

## Introduction

Irritable bowel syndrome (IBS) is a common chronic disorder affecting about 10–15% of the population in Western countries [1]. An IBS diagnosis is based on symptoms including abdominal pain and disturbed bowel function often related to and aggravated by psychosocial factors such as anxiety, depression and perceived stress [2]. Although it is a benign disorder, IBS is bothersome for patients and presents a burden to society due to its substantial effect on quality of life. In addition to the physical symptoms, the range of IBS-related difficulties for patients includes negative effects on socialization, family life, traveling and spontaneity due to the unpredictable bowel symptoms. On the societal level, IBS-related symptomatology leads to higher rates of work absenteeism and increased use of health resources [3]. The combined physical and social burdens associated with IBS highlight the importance of understanding how IBS impacts one’s own sense of health and interferes with functioning.

Self-rated health (SRH) is a distinct component of overall health [4] and a powerful predictor of clinical outcomes and mortality [5–7], even after controlling for objective ratings of health status [6]. SRH reflects the impact of psychological and social aspects of life and an overall sense of well-being [8]. It is widely used in epidemiological studies and typically is measured using a single question inquiring about the individual’s perception of their general health. Another important dimension of SRH is functional limitation, that is, the degree to which individuals experience that their health interferes with their life [9]. Self-reported functional limitations correlate with poor SRH, and both measures correlate with chronic health conditions [10, 11]. Although SRH is largely consistent with objective health status [11, 12], it also captures effects of other factors that co-act to influence perceptions of health. Examples include educational level, income, gender, psychosocial and emotional stress factors [6, 13, 14]. Several studies reveal SRH to be highly predictive of healthcare use, independent of the chronic health condition itself [15, 16]. For instance, Cislaghi et al. [15], showed that poor SRH, and not the presence of chronic somatic or mental conditions, was associated with the highest odds for hospital admission, number of specialist consultations and prescribed drugs. Only one large population-based study [17] has compared aspects of health-related quality of life between those with IBS versus chronic somatic diseases. Findings revealed that the IBS patients reported worse bodily pain, more fatigue, and worse emotional and social functioning.

Several studies of quality of life among IBS patients focus primarily on the negative effects of abdominal symptoms [18–20]. However, some investigations in the last two decades have used SRH as the measure of quality of life, and report that psychosocial factors and somatic comorbidity, rather than severity of symptoms, are the essential considerations affecting how IBS patients rated their overall health [21–23]. Furthermore, many IBS patients claim that consultations with gastroenterologists regarding their abdominal symptoms usually do not lead to improvements in their health [24]. Taken together, these results underscore the importance of a biopsychosocial or a holistic approach, rather than a symptom-driven approach, to understand the factors that underlie how IBS patients perceive their own health and their need of health care resources.

Only two large population-based studies have examined the association between IBS and SRH. These were from Denmark [25] and Canada [23], including 100,000 and 132,000 individuals, respectively. Both studies showed a greater likelihood of reporting poor SRH and functional limitations among those with IBS compared to the background population. The Canadian study is the only one to inquire about self-rated mental health in addition to general SRH, and revealed that IBS patients also reported poor mental health more often than the general population [23]. No studies have explored whether the perception of hindrance of daily activity in individuals with IBS is influenced by their perceptions of their physical or mental health.

The aims of this population-based twin study are to evaluate how IBS affects ratings of physical and mental health, and influences perceptions that daily activity are hindered by physical or mental health. An additional aim builds upon findings demonstrating that genetic factors contribute to variation in both SRH [26–29] and IBS [30–32]. However, to our knowledge, the genetic covariation between IBS and SRH measures has not been investigated previously. Therefore, we leverage the co-twin design to explore whether familial and/or genetic factors underlie the covariation between IBS and self-rated physical/mental health and between IBS and the perceptions of hindrance of daily activity by physical or mental health.

## Materials and methods

### Sample

The sample consists of 5288 twins aged 40–80 (average age 62.03, SD = 9.03) from the Norwegian Twin Registry [33]. In 2014–2015 the twins were invited to participate in a study on Social Factors and Health. They received a mailed questionnaire covering various aspects of their physical and mental health and social life, as well as their background information, including socio-economic status and relationship with the twin. This study is based on responses from twins from 1925 same-sex pairs of identical (MZ) and fraternal (DZ) twins, including 402 MZ and 375 DZ complete male pairs, 600 MZ and 548 DZ complete female pairs and 1438 single responders; 56.24% of the twins are female. A detailed description of the study, data and response rates are provided elsewhere [33].

### Measures

#### Demographic variables

*Income* was measured on a scale from one to nine reflecting the gross income of the total household in the last year. *Education* was measured based on the highest completed education, with seven alternatives, ranging from primary school (7–9 years) to PhD. Some response categories were collapsed based on the distribution of responses resulting in the following four categories: primary school (7–9 years); upper secondary school (1–3 years); college or university; advanced degree (e.g. M.D., PhD).

#### IBS

IBS status was determined using the question asking “Do you have irritable bowel syndrome (diarrhoea and/or constipation related to abdominal pain, at least once a week)?” The response alternatives were either “yes” or “yes, diagnosed by a doctor”. A positive reply to either of these options was coded as having IBS as supported by the analyses described below.

#### Self-rated physical and mental health

Self-rated physical (*p_SRH)* and mental health (*m_SRH)* were evaluated using separate questions asking: “How is your physical (mental) health at the moment?” with four available response categories: 1 = ’very good’, 2 = ’good,’ 3 = ’not so good’, 4 = ’bad’. On the basis of the distribution of responses, we then created two categories that combined “very good” and “good” into “good” SRH and “not so good” and “bad” into “poor” SRH.

#### Physical or mental health hinders activities

The measures of “physical/mental health hinders daily activities” were based on the following item: “How much have problems with your health (physical/mental) hindered you from social or work-related activities in the last four weeks?” The available response categories were: 1 = ’not at all’, 2 = ’a little’, 3 = ’some’, 4 = ’a lot’, 5 = ’very much’. These were also dichotomized based on the response distributions, by merging the first three categories into “no/little hindrance” and the last two categories, “a lot” and “very much” into “hindrance’’. The measures of physical and mental health interfering with the daily activities are labelled as *ph_hind* and *mh_hind*, respectively.

#### Pain

*Pain* is coded as a dichotomous (yes/no) measure based on information about whether the respondent experienced pain for any of the following pain-related conditions: durable and recursive muscle ache, neck pain, backache, pain in joints, migraine, headache and fibromyalgia. *Pain* was coded as positive if the respondent indicated they had experienced pain for at least one of these conditions or indicated ‘yes’ that the condition was diagnosed by a doctor.

#### Somatic diseases

A dichotomous measure reflecting the presence of somatic diseases (*Somatic*) was also constructed. This was based on questionnaire items asking whether the respondent had a history of any of the following chronic conditions and whether it was diagnosed by a doctor: asthma, cardio-vascular diseases (heart attack, hypertension, coronary heart disease), diabetes (I and II), autoimmune diseases (rheumatoid arthritis, Bechterew’s disease, psoriasis, AIDS), and cancer. Somatic diseases measure was assigned the value ‘1’, if at least one of these conditions was indicated “yes” or “yes, diagnosed by a doctor”.

#### Depression

Depression was based on the Center for Epidemiologic Studies Depression Scale (CESD-20) [34] with 20 items asking about twin’s condition in the last week. Items asked how often one was feeling bothered, depressed, fearful, lonely etc. The four response categories were: 0 = ’rarely/never’, 1 = ’few times’, 2 = ’quite many times’, 3 = ’almost all the time’. Positive items were reverse-coded, as recommended by the scale author, so that higher values correspond to higher levels of depression. The total CESD score was calculated only if four or less responses on the scale were missing, with the values imputed by the respondent’s mean.

#### Perceived stress

The Perceived stress scale (PSS) consists of 10 items asking about stress-related feelings and thoughts in the last month [35], e.g., how often did you feel upset due to unexpected events; how often did you feel like you were able to control important things in your life; how often did you feel anxious and nervous etc. The available response categories were: 0 = ’never’, 1 = ’almost never’, 2 = ’few times’, 3 = ’quite often’, 4 = ’very often’. Positive items were reverse-coded, so that a higher score indicates higher levels of perceived stress. Missing values were imputed by the respondent’s mean. The total PSS score was obtained by summing all the item responses for the cases with two or fewer missing values.

### Analysis

#### Merging IBS categories

Simple regression analyses controlling for age and sex were performed in order to test for the associations between the self-rated health measures with IBS in two different groups of responders: self-reported IBS and doctor diagnosed IBS. The estimates of the effect from IBS in these two groups were compared to each other and the same regression was run for the category of merged IBS responses. The analyses were conducted in the free statistical software R [36] by means of *geepack* package [37], which accounts for data dependency present in the twin data.

#### Descriptive statistics

Descriptive analyses were performed, including calculation of probandwise concordance rates for all the measures. Probandwise concordance is the proportion of “affected” twins whose twin is also “affected” in the sample of all “affected” twins, calculated as 2n_c_/(2n_c_ + n_d_), where n_c_ is the number of concordant pairs and n_d_ is the number of discordant pairs. In addition, correlations with age and sex, phenotypic, intraclass and cross-twin cross-trait correlations were computed for IBS and all health measures.

#### Hierarchical regressions

Hierarchical regressions were used to estimate the effects of IBS on perceptions of health, before and after accounting for other health conditions.

A set of six models was conducted for each of the response variables (*p_SRH*, *m_SRH*, *ph_hind*, *mh_hind*), where the following measures were added in a stepwise analysis: (m0) age + sex; (m1) m0 + *Income* + *Education*; (m2) m1 + IBS; (m3) m2 + *Pain*; (m4) m3 + *Somatic*; (m5) m4 + CESD + PSS. This procedure reveals whether the addition of the next set of predictor variables alters the significance of other measures in the model.

Each set of analyses was based on data from complete pairs for whom information was available on both twins for all the dependent measures and the response variable. Regressions were run by means of *glmer* function in *lme4* package in R [38]. Education and income were treated as continuous measures in these analyses.

#### Co-twin control analysis

Co-twin control analysis was employed to examine the nature of the relationships between IBS with the two measures of SRH (*p_SRH/m_SRH*) and with the two measures of degree to which health hinders daily activities (*ph_hind* /*mh_hind*). This approach helps to elucidate whether the relationships between an outcome (here measures of SRH) and an exposure (here IBS) are best explained by models of causality or by shared genetic or shared environmental effects [39, 40]. It is based on comparison of the odds ratios (ORs) of the outcome across three groups: IBS-discordant MZ pairs, IBS-discordant DZ pairs and unrelated individuals. Under a model of causality, we would expect that the OR for SRH (the outcome) is greater than 1 across all groups reflecting a higher risk of poor SRH among those with IBS. If pleiotropy (shared genes) explains the association between IBS and SRH, the OR in the general population will still be the same as under a causality model (> 1). However, among MZ pairs, we expect this OR to equal one because MZ twins share their genes and their genetic predisposition for poor health ratings and this would not be affected by IBS status. The respective OR for the outcome in the DZ pairs is expected to have an intermediate value between the unrelated and MZ estimates. The DZ twins share, on average, 50% of their genes and are more similar than persons in the general population, but less similar than MZ twins. Lastly, if the relationship is due to shared environmental factors that affect both the exposure and the outcome, then the OR for the outcome is expected to be equal to one among the MZ and DZ discordant pairs, as both members of the pair are equally exposed to the family environment that predisposes to both IBS and to SRH. In the general population, however, under this scenario IBS and poor health are still expected to be related with OR greater than 1. A more likely scenario may be that the relationship between IBS and SRH is only partly causal. Then we would expect the model to deviate with interpretations guided by the nature (common environment or genetic) of the effects.

This co-twin control analysis was conducted to analyze the relationships between IBS with each of the four health measures: *p_SRH*, *m_SRH*, *ph_hind*, *mh_hind*. All analyses were adjusted for age and sex effects. Each set of analyses was based on a subsample of complete pairs for whom data was available on both IBS, the respective health measure, age and sex. MZ and DZ groups consisted of twin pairs discordant for IBS, whereas the group of unrelated individuals was formed from all the single responders plus one random twin from each pair concordant for IBS. In order to get estimates of the ORs, generalized estimating equations (GEE) [41], were employed for the co-twin control analysis with the help of *geeglm* function in *geepack* package [37] in the free statistical software R [36]. Due to the small number of pairs in each dataset and hence wide confidence intervals, the OR comparison did not provide a clear picture of the nature of the analysed relationships and, therefore, three alternative models were tested against each other using OpenMx package [42] in R in order to decide which model (causal, shared genetic or shared environmental) best described each relationship. Model comparisons were based on Akaike’s Information Criterion (AIC), with lower AIC values indicative of a better fit.

## Results

In total, 348 twins (6.6%) reported IBS symptoms (self-reported), 21 twins (0.4%) answered “yes, diagnosed by a doctor”; 206 twins (3.9%) chose both options, self-reported and “diagnosed by a doctor”. The coefficients of the associations of the self-rated health measures with IBS did not differ significantly between self-reported and doctor diagnosed groups indicating that the two IBS classifications could be collapsed (Table 1). Merging the self-reported and doctor diagnosed groups resulted in 575 IBS cases (10.9%) available for the regression analyses of the association between IBS and SRH measures.

**Table 1:** Results comparing the associations of self-reported health measures with self-reported vs. doctor diagnosed IBS

Measure	Self-reported			Diagnosed			P est diff*	Combined		
	Estimate	SE	*p*	Estimate	SE	*p*		Estimate	SE	*p*
SRH physical										
Age	0.03	0.00	0.00	0.03	0.00	0.00	0.99	0.03	0.00	0.00
Sex	0.15	0.08	0.05	0.20	0.08	0.01	0.65	0.15	0.08	0.05
IBS	1.06	0.10	0.00	0.93	0.14	0.00	0.49	1.06	0.10	0.00
SRH mental										
Age	0.00	0.01	0.71	0.00	0.01	0.71	1.00	0.00	0.01	0.71
Sex	0.28	0.12	0.02	0.33	0.12	0.01	0.78	0.28	0.12	0.02
IBS	1.04	0.13	0.00	1.01	0.19	0.00	0.91	1.06	0.13	0.00
Physical health hinders activities										
Age	0.02	0.01	0.00	0.02	0.01	0.00	1.00	0.02	0.01	0.00
Sex	0.25	0.09	0.00	0.28	0.09	0.00	0.78	0.25	0.09	0.00
IBS	0.88	0.11	0.00	1.06	0.15	0.00	0.34	0.89	0.11	0.00
Mental health hinders activities										
Age	0.02	0.01	0.02	0.02	0.01	0.02	1.00	0.02	0.01	0.02
Sex	0.20	0.12	0.10	0.26	0.12	0.04	0.74	0.20	0.12	0.10
IBS	1.13	0.14	0.00	1.10	0.20	0.00	0.90	1.12	0.14	0.00

*p-value for a difference between estimates in the self-reported group and the doctor diagnosed group

Of the twins who report poor health, 18.8% report poor physical health and 14.5% report that physical health hinders their daily activity. The corresponding values for the measures of mental health were 7.3% and 6.5%. Table 2 includes information on the frequency, probandwise concordance rates and interclass correlations of IBS and health rating measures by sex and zygosity.

**Table 2:** Number of cases, concordant measures and intraclass correlations for the study measures

Measure	Cases, N (%)			Concordant* pairs, n (%)				Discordant **pairs, n (%)		Probandwise concordance rate, % (95% CI)		Intraclass correlations (95% CI)	
	Total	m	f	MZ		DZ		MZ	DZ	MZ	DZ	MZ	DZ
				++	− −	++	− −						
SRH													
Physical	987 (18.8)	396 (17.2)	591 (20.1)	81 (8.1)	677 (67.6)	33 (3.6)	618 (67.0)	225 (22.5)	256 (27.7)	41.2 (35.7;48.0)	20.5 (14.6;26.4)	0.48 (0.43;0.52)	0.07 (0.01;0.13)
Mental	376 (7.3)	134 (6.0)	242 (8.4)	17 (1.7)	821 (81.9)	6 (0.7)	753 (81.6)	115 (11.5)	101 (10.9)	22.8 (13.8;31.8)	10.6 (2.8;18.4)	0.39 (0.33;0.44)	0.14 (0.08;0.20)
Health hinders activities													
Physical	742 (14.5)	281 (12.6)	461 (16.1)	42 (4.2)	699 (69.8)	26 (2.8)	645 (69.9)	188 (18.8)	187 (20.3)	30.9 (23.7;38.0)	21.8 (14.8;28.7)	0.38 (0.32;0.43)	0.20 (0.14;0.26)
Mental	326 (6.5)	123 (5.6)	203 (7.2)	49 (4.9)	786 (78.4)	5 (0.5)	738 (80.0)	106 (10.6)	76 (8.2)	11.7 (3.8;19.5)	11.6 (2.3;20.9)	0.17 (0.11;0.23)	0.23 (0.17;0.29)
IBS	575 (10.9)	174 (7.5)	401 (13.5)		29 (2.9)		15 (1.6)	168 (16.8)	175 (19.0)	25.7 (18.1;33.2)	14.6 (8.0;21.2)	0.35 (0.29;0.40)	0.10 (0.04;0.17)

*A twin pair was considered to be concordant for a health measure if both twins scored either 0 or 1 on the respective measure.

**Discordance for SRH and the measures of health interfering with daily activities was based on the respective dichotomized measures

### The effect of sex and age on IBS and health rating measures

Correlations of the health rating measures with sex were small, (between 0.07 and 0.11), but significant (*p* < 0.05). Women tended to report worse physical and mental health than men. IBS correlated moderately with sex (0.20 [0.15;0.26]), reflecting a greater prevalence among females than males (13.5% vs. 7.5%). Older age was significantly correlated with *p_SRH* (0.12 [0.08;0.15]), but there was no age affect for *m_SRH* (0.01 [− 0.04;0.06]), older age was also associated with a greater degree of health hindering effects for *ph_hind* (0.08 [0.04;0.11]) and *mh_hind* (0.06 [0.01;0.11]).

### Intraclass correlations and probandwise concordance rates

The probandwise concordance rates and intraclass correlations for IBS and the SRH measures were greater among MZ compared to DZ pairs, except for *mh_hind* (Table 2).

This pattern is consistent with genetic effects influencing variation in these measures. For the measure *mh_hind,* the magnitude of these estimates did not differ between MZ and DZ twins*,* which suggests that that shared environment may explain the variance of this measure.

### Phenotypic and cross-twin cross-trait correlations

**Fig. 1:**
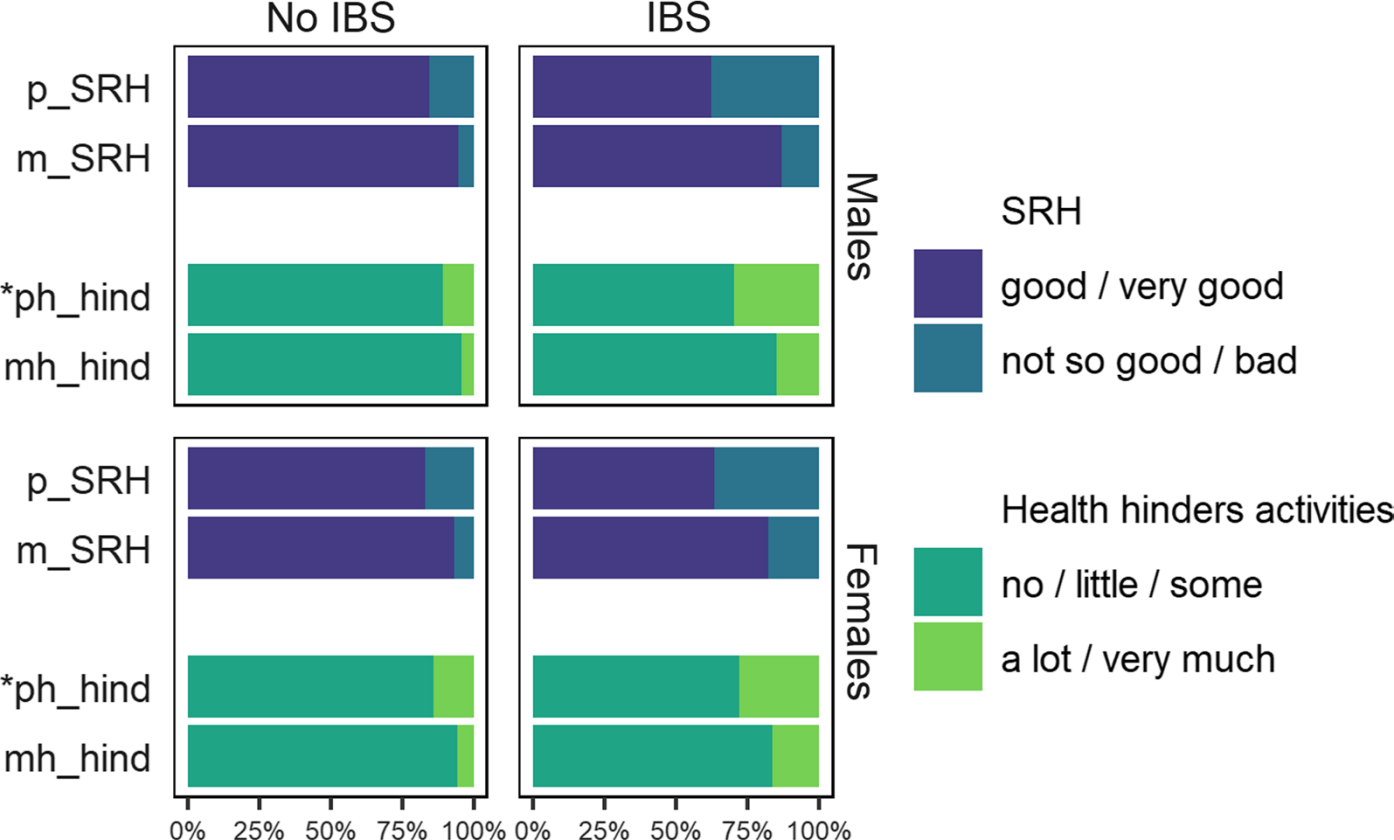
Distribution in percentiles of responses to the self-reported health measures by sex and IBS status. *Note*: * significant (*p* = 0.01) sex differences for twins with no IBS

Twins with IBS tend to rate their health as worse or report that their health hinders their daily activities to a greater degree than twins without IBS (Fig. 1). The health rating measures did not vary significantly by sex, however, among twins without IBS, more women than men reported that their physical health constrains daily activities.

**Fig. 2:**
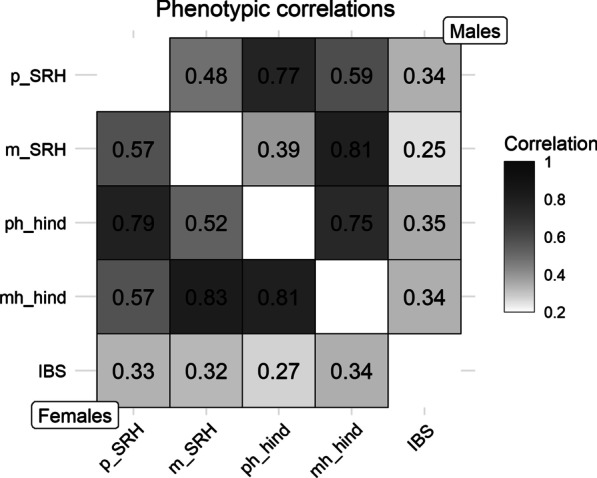
Phenotypic correlations between the study measures by sex. *Note*: All correlations were significant with *p* < 0.01

Figure 2 displays a heatmap of the phenotypic correlations by sex. Correlations were significant for all pairs of measures, with the highest values between self-rated health measures (*p_SRH*, *m_SRH*, *ph_hind*, *mh_hind*) and the lowest values for the correlations between IBS and the self-rated health measures.

Cross-twin cross-trait correlations (Table 3) between IBS and the self-rated health measures were similar across zygosity, except for IBS – *m_SRH* relationship. This correlation was significantly greater in MZ compared to DZ pairs (0.23 vs. 0.04), suggesting that the association between IBS and self-rated mental health is primarily explained by shared genes.

**Table 3:** Cross-twin cross-trait correlations and 95% CI for the study by zygosity

IBS correlation with	MZ	DZ
SRH physical	0.13 (0.09;0.18)	0.16 (0.11;0.20)
SRH mental	0.23 (0.19;0.27)	0.04 (− 0.01;0.09)
Physical health hinders activities	0.19 (0.15;0.23)	0.16 (0.11;0.20)
Mental health hinders activities	0.15 (0.11;0.20)	0.14 (0.10;0.19)

### Hierarchical regression models

**Fig. 3:**
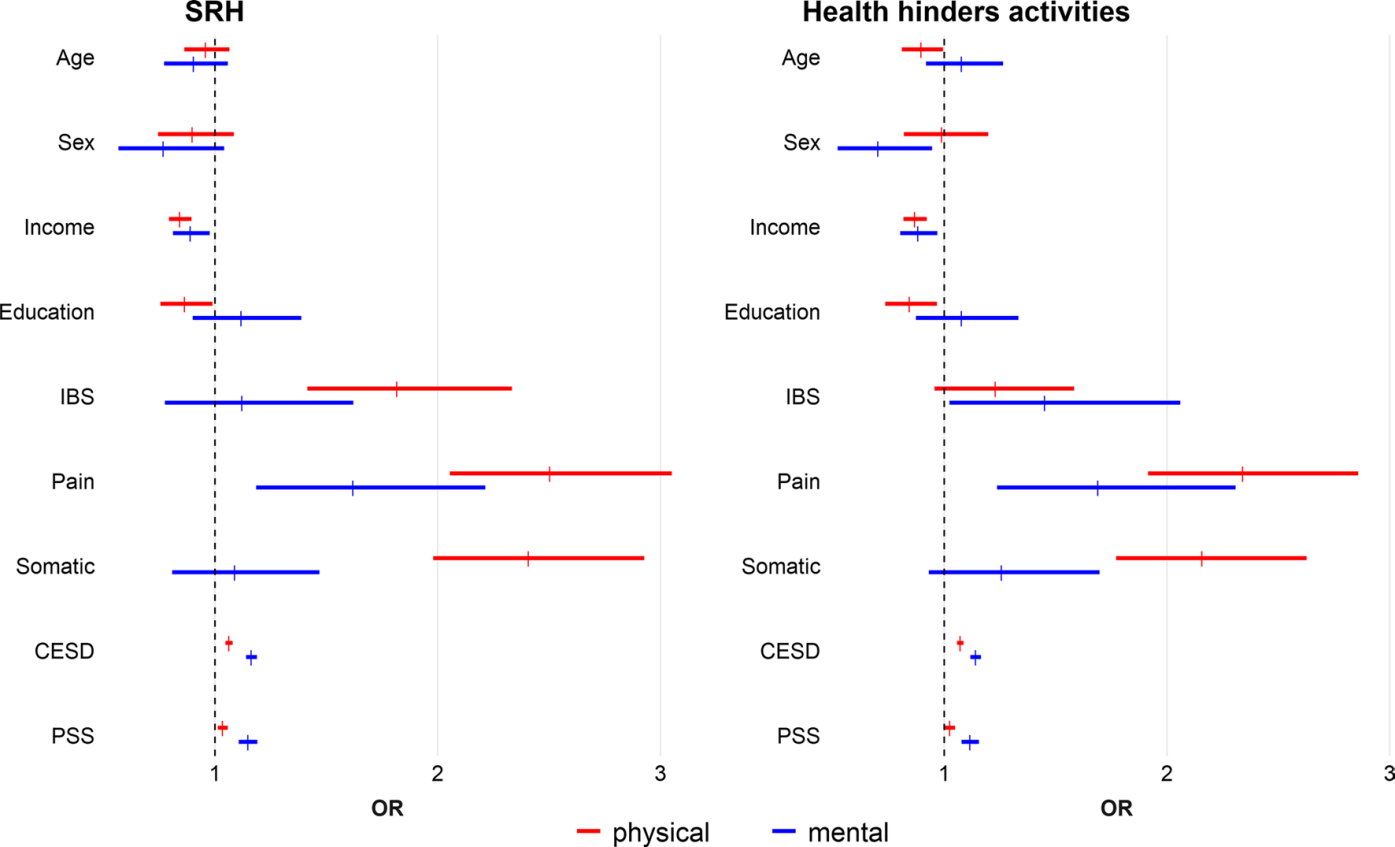
OR’s and 95% CI from model m5. *Note*: The estimates are controlled for age, sex, income, education and health conditions

Table 4 presents results from the hierarchical regression analyses and Fig. 3 displays the ORs of poor SRH and hindrance of daily activities from final results of the regression analyses (model m5). After adjusting for the other measures in the model, age and sex effects were not predictive of the outcomes with the exception of age for *ph_hind* and sex for *mh_hind.* These findings indicated that older people were less likely to report hindrance of their daily activities by their physical health, and women were likely to report less hindrance of daily activities by their mental health. Income was inversely associated with all the health rating measures, whereas education retained significance only for the measures related to physical health with higher education predictive of better perceptions of health and less hindrance of daily activities.

**Table 4:** Results from regression models showing effects of covariates on self-reported health measures

Measure	Physical SRH						Mental SRH					
	m0	m1	m2	m3	m4	m5	m0	m1	m2	m3	m4	m5
Age	1.26***	0.97	0.99	0.98	0.90*	0.96	0.99	0.77***	0.77***	0.77***	0.74***	0.90
Sex	1.30**	1.08	1.01	0.94	0.98	0.90	1.43	1.17	1.09	1.05	1.06	0.77
Income		0.76***	0.79***	0.78***	0.78***	0.84***		0.71***	0.72***	0.73***	0.73***	0.89*
Education		0.74***	0.78***	0.79***	0.80**	0.86*		0.82*	0.84*	0.87	0.87	1.12
IBS			2.59***	2.41***	2.33***	1.82***			2.45***	2.16***	2.10***	1.12
Pain				2.82***	2.72***	2.50***				1.93***	1.88***	1.62**
Somatic					2.55***	2.41***					1.42**	1.09
CESD						1.06***						1.16***
PSS						1.04**						1.15***

Measure	Physical health hinders activities						Mental health hinders activities					
	m0	m1	m2	m3	m4	m5	m0	m1	m2	m3	m4	m5
Age	1.15**	0.91*	0.90	0.91	0.854***	0.90*	1.17	0.87*	0.88	0.89	0.85*	1.08
Sex	1.37***	1.16	1.14	1.05	1.08	0.99	1.26	1.06	0.98	0.94	0.95	0.70*
Income	–	0.78***	0.76***	0.79***	0.80***	0.87***	–	0.70***	0.71***	0.72***	0.73***	0.88**
Education	–	0.74***	0.69***	0.78***	0.79***	0.84*	–	0.83*	0.84	0.87	0.88	1.08
IBS	–	–	2.33***	1.71***	1.63***	1.23	–	–	2.83***	2.48***	2.40***	1.45*
Pain	–	–	–	2.62***	2.52***	2.34***	–	–	–	2.02***	1.97***	1.69***
Somatic	–	–	–	–	2.31***	2.16***	–	–	–	–	1.56*	1.26
CESD	–	–	–	–	–	1.07***	–	–	–	–	–	1.14***
PSS	–	–	–	–	–	1.03*	–	–	–	–	–	1.12***

OR’s larger than 1 reflect increased odds for ‘poor’ SRH and ‘a lot’/’very much’ hindrance of activities

***p < 0.001; **p < 0.01; *p < 0.05

Pain, depression (CESD) and perceived stress (PSS), were predictive of all four health measures. Somatic conditions were significant only for the physical health measures.

### The impact of IBS on the health rating measures

The effect of IBS retained significance for all the health rating measures in all models up to m4; however, after accounting for the effects of depression (CESD) and perceived stress (PSS), IBS remained an important predictor for *p_SRH* and *mh_hind* (m5 in Table 4).

The association between poor *p_SRH* and IBS was comparable to the associations between poor *p_SRH* and chronic somatic conditions (Fig. 3). However, in contrast to chronic somatic conditions, IBS was associated with the perception that mental health and not physical health interfered with daily activity.

### Familial effects and the covariance between IBS and perceptions of health

**Fig. 4:**
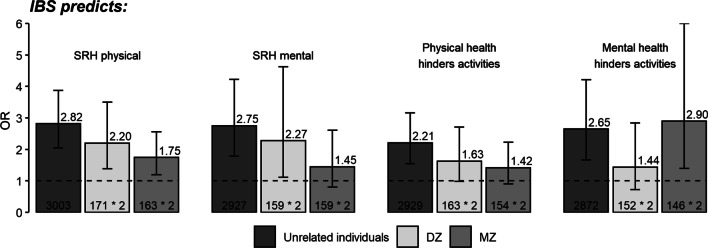
Results of the co-twin control analysis testing for causality vs pleiotropy

Results of the co-twin control analysis are depicted in Fig. 4. OR’s for the relationship between IBS and *p_SRH* were significantly greater than 1.0 in all three groups (MZ, DZ and unrelated individuals). This pattern corresponds to a causal model explaining the relationship between these two measures. Results for the relationship between IBS and *m_SRH* were consistent with the pleiotropy model whereby shared genes explain this relationship.

Results for the relationship between IBS and hindrance of daily activities by physical health (IBS – *ph_hind*) were most consistent with a model of shared environment underlying the association. Analysis of the relationship between IBS and *mh_hind* did not yield a clearly interpretable pattern of OR results across the three groups.

Table 5 presents the results comparing the fit of alternative models of the relationships between IBS and the health rating measures. The findings indicate that the causality model is the best model explaining the associations between IBS and *p_SRH*, between IBS and *ph_hind* and between IBS and *mh_hind*. For the IBS—*m_SRH*, the relationship seems to be driven by common genes, which is in agreement with tetrachoric correlations and the results from the co-twin control analysis above.

**Table 5:** Comparative fits of models to explain the relationships between IBS and self-rated health measures

	ep	− 2LL	df	AIC	χ^2^	Δdf	*p*
*IBS predicts*							
SRH physical							
Saturated	17	3589.51	3741	− 3892.49	–	–	–
Causal	11	3595.77	3747	− **3898.23**	6.26	6	0.40
Genetic	13	3599.89	3745	− 3890.11	10.38	4	0.04
Shared environmental	11	3610.43	3747	− 3883.57	20.92	6	0.00
SRH mental							
Saturated	17	1935.29	3628	− 5320.71	–	–	–
Causal	11	1944.93	3634	− 5323.07	9.64	6	0.14
Genetic	13	1938.01	3632	− **5325.99**	2.72	4	0.61
Shared environmental	11	1947.56	3634	− 5320.45	12.26	6	0.06
Physical health hinders activities							
Saturated	17	3017.39	3626	− 4234.61	–	–	–
Causal	11	3020.16	3632	− **4243.85**	2.76	6	0.84
Genetic	13	3020.88	3630	− 4239.12	3.48	4	0.48
Shared environmental	11	3024.38	3632	− 4239.63	6.98	6	0.32
Mental health hinders activities							
Saturated	17	1737.35	3539	− 5340.65	–	–	–
Causal	11	1743.52	3545	− **5346.48**	6.17	6	0.41
Genetic	13	1747.07	3543	− 5338.93	9.72	4	0.05
Shared environmental	11	1750.73	3545	− 5339.27	13.38	6	0.04

*ep* estimated parameters; *− 2LL* minus to log-likelihood; *df* degrees of freedom; *AIC* Akaike’s Information Criterion; *χ*^*2*^ difference in the log-likelihood to the base model; *Δdf* difference of the degrees of freedom with the base model; *p* *p*-value. In bold—the lowest AIC corresponding to the best fitting model for each of the relationship

## Discussion

The present study explores how IBS affects ratings of physical and mental health, and influences perceptions of hindrance of daily activity by physical or mental health. Further, we investigate whether the relationships between IBS and self-ratings of health are best explained by a model of causality or rather reflect common underlying familial and/or genetic factors. To our knowledge, this is the first study to explore family effects, genetic or shared environmental effects, underlying the relationship between IBS and SRH measures.

Our main findings reveal that IBS is predictive of poor ratings of physical health, and further, that mental health, and not physical health, are perceived to interfere with daily activities. The co-twin analyses suggest that causal mechanisms best explained the relationships between IBS with self-rated physical health and with the perception that health interferes with daily activity. Further, both the co-twin control design and the correlation analyses provide evidence that genetic effects are shared between IBS and self-rated mental health.

### IBS prevalence

The prevalence of IBS was 10.9%, which is similar to the rates in most European countries [43]. The prevalence was almost twice as high among females than males (13.5% versus 7.5%), consistent with most studies [44, 45]. Further, 39.5% of the IBS cases were doctor-diagnosed, similar to rates reported in some previous studies [46, 47].

### The impact of IBS on ratings of self-rating health measures

The present study assessed two dimensions of SRH, physical and mental, and inquired about the extent to which physical or mental health interfered with daily activities.

Our findings revealed that individuals with IBS reported worse physical health compared to those without IBS (OR = 1.82, 95% CI [1.41;2.33]).

Studies comparing self-reported quality of life among individuals reporting IBS with those who report other chronic somatic conditions are scarce. One study from California University compared the health-related quality of life (HRQOL) of 877 IBS patients enrolled from a large tertiary referral centre with previously published data from the general population and from an observational study including individuals with chronic diseases such as diabetes, end-stage renal disease and depression [17].

Individuals with IBS scored lower on all the HRQOL measures: energy/fatigue, bodily pain, emotional and social function, except for physical function, compared to those who suffered from the above mentioned chronic somatic conditions. Further, those who suffered from depression had worse physical functioning caused by emotional and psychosocial health problems than IBS-sufferers. However, it is worth noting that it was not possible to control for age, gender, race, education or comorbidity in these analyses, because the IBS patients and those who suffer from chronic somatic diseases or depression, stem from different studies.

Results from the hierarchical regression analyses in our population-based study, revealed that the association between IBS and poor *p_SRH* was comparable with the association between somatic conditions and poor *p_SRH* (OR = 2.41, CI 95% [1.98; 2.93], which underscores the potential debilitating effects of IBS.

The association between IBS and self-rated mental health was confounded only by PSS and depression. The confounding effects are explained by the strong association between *m_SRH* and depression or PSS, and between IBS and depression [48] or PSS [49]. Stress is an important environmental factor in the pathophysiology of IBS which affects various aspects of IBS, including disease onset or exacerbation of abdominal symptoms among individuals already suffering from IBS.

Environmental experiences common to IBS, PSS and depression such as restricted uterine growth, traumatic events or chronic stressors in early life and /or adulthood [50–53] are all triggers of the central stress pathways, the sympathetic nervous system and the hypothalamic–pituitary–adrenal (HPA) axis. Twin studies have provided evidence that shared genetic effects help to explain the covariation between IBS and depression [52] and between IBS and social stressors [54], suggesting that these disorders share genes involved in central stress mechanisms. Results from candidate studies also suggest that depression [55, 56] and perceived stress [57, 58] share in common gene variants with IBS.

Only two population-based studies [23, 25] have explored the impact of IBS on self-rated health. Both studies used a single question inquiring about the individual’s perception of their general health and one for functional limitations. The studies demonstrated that participants with IBS rate their overall health and their functional capacity worse compared to the general population. Tang et al. [23] also included self-rated mental health and the prevalence of other chronic conditions among those with IBS. Their findings [23] were consistent with our results showing that mental disorders affect IBS ratings of mental health among those with IBS. They report that that the odds for poor ratings of mental health were significantly higher among individuals with IBS and comorbid mental disorders (anxiety, mood disorder and other) than those who suffer from only IBS.

### The impact of IBS on the perception of hindrance of daily activity by health

Perceptions of health, are strongly associated with physical functioning and both measures are linked to chronic health conditions [15]. For most patients with chronic conditions, it is their ability to function in their daily activities that matters. Therefore, it is important to elucidate the factors that shape the experiences of and perceptions of IBS patients of how their health hinders their daily activities.

To our knowledge, our study is the first to investigate whether individuals with IBS experience that their physical or mental health hampers their daily activity.

Although IBS was strongly associated with poor ratings of physical health, individuals with IBS report interference of daily activities by their mental health, and not by their physical health, after accounting for PSS and depression.

These findings suggest that self-rated physical and mental health are multidimensional constructs, influenced by other factors than IBS symptoms and depression or PSS, respectively.

Lackner et al. [21] emphasized the importance of fatigue, psychosocial and emotional factors when individuals with IBS rate their health.

In contrast to those with IBS only, those with other chronic somatic conditions reported interference of daily activity only by physical health (Fig. 3).

The link between IBS and mental health interfering with daily activity is consistent with several studies [21, 22, 24, 59] showing that psychosocial, emotional and social factors were more important than severity of physical symptoms when IBS patients rate their quality of life. For instance, Weert et al. [60] report that decreased severity of symptoms did not impact quality of life. About 30% of patients who no longer fulfil the Rome III criteria after a 5‐year follow‐up period, did not have improved quality of life.

### The relationship between IBS and self-reported health measures: causality or shared genetic pathways—co-twin control analyses

The co-twin control design seeks to discriminate causal from non-causal relationships, in this study, between IBS as a predictor and all the SRH measures. Our findings suggest that a causal model best explained the relationships between IBS and self-rated physical health and between IBS and the extent to which physical or mental health hinders daily activity. In contrast, the relationship between IBS and self-rated mental health most likely reflects the effects of shared genetic factors (Fig. 4) which might in part, explain the covariation between IBS and the extent to which mental health interferes with functional impairment. These results are consistent with the correlation analyses showing a greater correlation between IBS and self-rated mental health among MZ compared to DZ twins. These analyses do not explore shared genetic or shared environment between IBS and covariates such as depression, stress and psychosocial or emotional factors, but analyse the association between two complex traits, self-rated mental health and IBS. However, the finding of genetic influences for IBS implicate a broad array of mechanisms from the brain to the gut, involving central processing, immune function and visceral sensitivity in interaction with the HPA-axis [43]. Psychosocial stressors, personality traits and emotional state are all factors that influence these mechanisms, in part through shared genetic pathways [52, 54, 61, 62].

Kutschke et al. [54] demonstrated that genetic variation of IBS was fully shared with social stress factors, like social strain and low support in close relationships, suggesting that genes involved in central stress mechanisms are the main source of the genetic variation of IBS.

Twin studies have revealed that genetic effects contribute to the variation of IBS [30–32] as well as to SRH and functional limitation dependent on age and sex [26, 28]. The study of Leionen et al. [28]-demonstrated that SRH shared genetic effects with functional limitation, severity of disease and depression, which accounted for 64% of the genetic variation of SRH. Our study was underpowered to perform similar analyses, but the co-twin control analyses and analyses of alternative models testing for causality, shared genes (pleiotropy) or shared environment (Table 5), suggested that the relationship between IBS and self-rated mental health seems to be explained by common genes.

## Limitations

The main limitation of this study is the lack of power, due to sample size. Unfortunately, only twins aged between 40–80 years were invited to the twin study in 2014–2015, partly because of the purpose of the study, exploring the association between social factors and health, and partly because the younger age group, 20-40 years, was not included in the Norwegian twin registry until 2021.

Although the analyses included 575 cases of IBS, the sample size restricts the ability of our analytical models to differentiate between shared environmental effects and genetic effects in the co-twin control analyses, especially for whether IBS predicts hindrance of daily activity by physical or mental health. However, additional analyses, the fit statistics of the co-twin control analyses, contributed to the final interpretation of the results. The fit statistics, the comparisons between alternative models in order to decide which model best described each relationship, indicate that the causality model is the best model explaining the associations between IBS and *ph_hind*, between IBS and *mh_hind*.

Another limitation is that most of the IBS diagnoses were self-reported. We used a short version of Rome IV criteria [63]: “Do you have or have you ever had IBS including abdominal pain and disturbed bowel functions, constipation and/or diarrhoea, at least once a week”. The response alternatives were either “yes” or “yes, diagnosed by a doctor. They did confirm current symptoms, but since we did not ask them about age of onset of symptoms, we were not able to consider the chronic course of intermittent abdominal symptoms typical for IBS. However, they selectively endorsed the question of IBS among 42 additional health problems and diseases which suggests that they were familiar with the term.

Although the Rome III criteria was still valid in 2014–2015, when we mailed out the questionnaire, we chose the short version of the Rome IV criteria for the IBS symptoms, knowing that these criteria are more restrictive than the Rome III. We wanted to ensure the IBS diagnosis and to include individuals with as homogeneous symptoms as possible. Approximately 39,5% of the twins who have reported IBS symptoms, were also diagnosed by a doctor. Importantly, the coefficients of the associations between self-rated health measures and IBS, did not differ between doctor-diagnosed IBS or self-reported IBS (Table 1).

Warning symptoms of IBS were not included in the questionnaire of the twin study “Social factors and health” in 2014–2015. The main aim of that study was to explore the association between social life and health, and IBS was listed among 42 other disorders/diseases in the questionnaire. However, the positive predictive value of warning symptoms for the IBS diagnosis is disputable. Yang et al. [64], using Rome IV criteria for IBS, did not find any significant improvement of the positive predictive value of the IBS diagnosis when including alarm symptoms.

A small number of individuals with IBS had concomitant coeliac disease (1.6%), Crohn’s disease (CD) (1.9%) and ulcerative colitis (UC) (2.6%). Individuals with IBS have a higher risk of coeliac disease [65] or UC and CD in clinical and endoscopic remission [66] compared to the general population. In the present study, the prevalence of CD and UC were significantly higher (*p* = 0.021 and *p* = 0.022, respectively) among individuals with IBS (self-rated or doctor-diagnosed) compared to those without IBS. Since the above-mentioned diagnoses always involve doctor consultation including endoscopies, we believe these IBS cases suffer from both IBS and the comorbid conditions.

## Conclusion

Our results show that having IBS was predictive of reporting poor physical health and the effect size of IBS on ratings of physical health was comparable to chronic somatic diseases. However, in contrast to somatic diseases, IBS was associated with the extent to which mental, and not physical health, interferes with daily social and work-related activities.

It is essential to communicate with IBS patients about their perceptions of quality of life, and possible underlying factors of reduced quality of life, to offer proper and effective treatment.

According to our results, psychosocial and emotional factors, are important when IBS patients perceive their physical and mental health. Clinicians should consider a biopsychosocial approach towards their IBS patients, rather than a symptom driven approach, to uncover determinants of their perceptions of health. Further, the choice of treatments should reflect this, by for example treatment strategies oriented towards stress reaction and reduction such as hypnotherapy and mindfulness treatment.

## Data Availability

The consent given by the participants does not open for storage of data on an individual level in repositories or journals. Researchers who want access to data sets for replication should submit an application to datatilgang@fhi.no. Access to data sets requires approval from the Regional Committees for medical and health research ethics in Norway and a formal contract with The Norwegian Twin Registry.
